# *Mycobacterium tuberculosis *and *Clostridium difficille *interactomes: demonstration of rapid development of computational system for bacterial interactome prediction

**DOI:** 10.1186/2042-5783-2-4

**Published:** 2012-03-21

**Authors:** Seshan Ananthasubramanian, Rahul Metri, Ankur Khetan, Aman Gupta, Adam Handen, Nagasuma Chandra, Madhavi Ganapathiraju

**Affiliations:** 1 Department of Biomedical Informatics, University of Pittsburgh, Pittsburgh 15260, USA; 2 Intelligent Systems Program, University of Pittsburgh, Pittsburgh 15260, USA; 3 Department of Biochemistry, Indian Institute of Science, Bangalore 560012, India; 4 Indian Institute of Technology, Roorkee, India; 5 Birla Institute of Technology and Science, Pilani, India; 6 Rochester Institute of Technology, Henrietta, USA

## Abstract

**Background:**

Protein-protein interaction (PPI) networks (interactomes) of most organisms, except for some model organisms, are largely unknown. Experimental methods including high-throughput techniques are highly resource intensive. Therefore, computational discovery of PPIs can accelerate biological discovery by presenting "most-promising" pairs of proteins that are likely to interact. For many bacteria, genome sequence, and thereby genomic context of proteomes, is readily available; additionally, for some of these proteomes, localization and functional annotations are also available, but interactomes are not available. We present here a method for rapid development of computational system to predict interactome of bacterial proteomes. While other studies have presented methods to transfer interologs across species, here, we propose transfer of computational models to benefit from cross-species annotations, thereby predicting many more novel interactions even in the absence of interologs. *Mycobacterium tuberculosis *(Mtb) and *Clostridium difficile *(CD) have been used to demonstrate the work.

**Results:**

We developed a random forest classifier over features derived from Gene Ontology annotations and genetic context scores provided by STRING database for predicting Mtb and CD interactions independently. The Mtb classifier gave a precision of 94% and a recall of 23% on a held out test set. The Mtb model was then run on all the 8 million protein pairs of the Mtb proteome, resulting in 708 new interactions (at 94% expected precision) or 1,595 new interactions at 80% expected precision. The CD classifier gave a precision of 90% and a recall of 16% on a held out test set. The CD model was run on all the 8 million protein pairs of the CD proteome, resulting in 143 new interactions (at 90% expected precision) or 580 new interactions (at 80% expected precision). We also compared the overlap of predictions of our method with STRING database interactions for CD and Mtb and also with interactions identified recently by a bacterial 2-hybrid system for Mtb. To demonstrate the utility of transfer of computational models, we made use of the developed Mtb model and used it to predict CD protein-pairs. The cross species model thus developed yielded a precision of 88% at a recall of 8%. To demonstrate transfer of features from other organisms in the absence of feature-based and interaction-based information, we transferred missing feature values from Mtb orthologs into the CD data. In transferring this data from orthologs (not interologs), we showed that a large number of interactions can be predicted.

**Conclusions:**

Rapid discovery of (partial) bacterial interactome can be made by using existing set of GO and STRING features associated with the organisms. We can make use of cross-species interactome development, when there are not even sufficient known interactions to develop a computational prediction system. Computational model of well-studied organism(s) can be employed to make the initial interactome prediction for the target organism. We have also demonstrated successfully, that annotations can be transferred from orthologs in well-studied organisms enabling accurate predictions for organisms with no annotations. These approaches can serve as building blocks to address the challenges associated with feature coverage, missing interactions towards rapid interactome discovery for bacterial organisms.

**Availability:**

The predictions for all Mtb and CD proteins are made available at: http://severus.dbmi.pitt.edu/TB and http://severus.dbmi.pitt.edu/CD respectively for browsing as well as for download.

## Background

The presence of about 500-1,000 bacterial species in the human gut flora of the intestines plays important role in immunity and nutrition [1]. While some bacteria live in a symbiotic relationship with humans, numerous others cause diseases, killing millions of people annually. *Mycobacterium tuberculosis *(Mtb) causes tuberculosis which remains a leading infectious disease to this day, with about 2 million deaths annually worldwide [2-4]. A deadly synergy with human immunodeficiency virus (HIV) further increases the burden of the disease [5,6]. *Clostridium difficile *(CD) infection is the primary cause of antibiotic-associated diarrhoea. It has a property of undergoing mutation rapidly [7]. In the past ten years, variant toxin-producing strains of *C. difficile *have emerged, that have been associated with severe disease outbreaks worldwide [8]. Understanding the functions of the proteins of the pathogens, and their interactions with each other and with host proteins would provide a basis for understanding the pathogenesis and virulence mechanisms of the pathogen [9-11]. Analyses of protein-protein interaction (PPI) networks have led to rich insights about possible pathways of information flow that could lead to the emergence of drug resistance in the pathogen [12]. A better understanding of pathogen-interactome can also help in studying host-pathogen interactions [13,14]. Despite the importance of PPIs, the high cost, time-consuming and labor intensive nature of experimental methods have resulted in limited availability of currently known PPIs.

Newer techniques such as mass-spectrometry, yeast two-hybrid (Y2H), and tandem affinity purification have been designed to study the global interactions in an organism in a high-throughput manner. Y2H methods have a low recall, in the order of about 12% [15]. This low recall, coupled with the fact that only one in a thousand pairs of proteins is an interacting pair, makes it expensive as well as infeasible to characterize whole interactomes with only high throughput biotechnology. Data explosion in the post-genomic era has resulted in advancement in computational methods to extract and analyze useful biological information, including PPIs. Computational methods for PPI prediction use biological information such as genomic data, 3D structures, gene co-expression, co-occurrence, co-evolution, and co-localization as features based on which a model is learnt to classify interacting pairs from non-interacting pairs [16]. Although computational methods are being developed for discovering more and more new PPIs in organisms, the required feature-base (gene expression profiles, gene ontology annotations, etc) is not available except for a few well studied organisms such as yeast, mouse and human. For other organisms for which a rich feature-base is not available, some PPIs may be inferred *bioinformatically *by identifying interactions among orthologs (namely, by identifying *interologs*) [17,18] or with computational methods that use only protein sequence information. Phylogenetic profiling infers protein interactions from patterns of presence or absence of proteins across multiple genomes. If genes are functionally related in an organism, co-inheritance of the genes would occur in other organisms, since the absence of any of the genes would result in the loss of function of the other genes [19]. Gene fusion predicts interactions between two proteins in an organism based on the evidence that they form a part of a single protein in a different genome [19]. Gene order conservation infers interactions based conserved neighbourhood of genes on the genome [20]. Similarity of Phylogenetic trees assumes that interacting protein sequences and their partners must co-evolve and pairs of protein sequences exhibiting high degree of co-evolution are inferred to be interacting [21]. Some methods adopt sequence-based approaches, which include sequence co-evolution, a method based on multiple sequence alignments and domain pairs-based approaches, where domain-domain interactions are noted to be conserved across species [22]. Homology-based approaches are based on both sequence and structure information to transfer known interactions [23,24]. Some studies have used model organisms to aid in the discovery of the interactome of another organism. Using domain-domain interactions, it has been shown that it may be significantly more precise to predict PPIs from multiple organisms than from a single organism [25]. *Streptococcus pneumoniae *interactome determined with Y2H was compared with that of *E. coli*, and a large number of conserved interactions were found among the orthologs between the two organisms [26].

**Figure 1:**
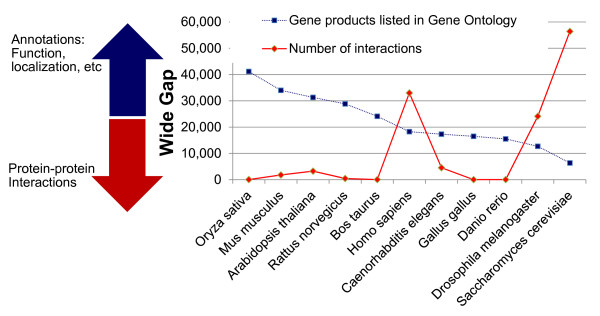
**Wide-gap in the availability of annotations and protein-protein interactions**. Wide-gap in the availability of Gene Ontology annotations and protein-protein interactions. The blue line shows the number of proteins whose annotations are available in Gene Ontology () [1] and the red line shows the number of interactions available in BioGrid (thebiogrid.org) [2] for the organisms.

With any of these methods, only a few potential interactions are discovered, making it necessary to devise other methods for discovering more of the hitherto-unknown interactions. In this work, we demonstrate that cross-species transfer of information may be carried out not only bioinformatically, but also by transfer of computational models across organisms. We show that a machine learning model developed for one organism may also be applied to predict PPIs of another organism based on the former's model and the latter's feature set. We used two specific pathogens *Mycobacterium tuberculosis *(Mtb) and *Clostridium difficile *(CD) to demonstrate the method. The new interactions predicted for these two organisms are also made available. For this work, we selected STRING and GO data as features for our prediction method as these features are available for a large number of organisms. STRING database provides biophysical and functional interactions among proteins for 869 bacterial organisms [27]. It gives a score of functional association for protein pairs computed based on their gene co-occurrence, co-expression, gene fusion, gene neighbourhood, experimental validation, text mining data and data from other databases, individually and cumulatively scored. GO provides annotation to 1,209 bacterial organisms. Figure 1 shows the wide-gap in the availability of interactions compared to the availability of function, localization and biological process membership of proteins. The x-axis shows the ten organisms which have the most annotations and interactions known to-date. This plot clearly shows that the annotations like function and localization are known in much larger numbers for most organisms, whereas only very few PPIs are known (data from Gene Ontology [1], http://www.geneontology.org and BioGrid [28,29], thebiogrid.org).

## Results and discussion

Predicting PPIs is treated as a classification problem in machine learning. Each protein-pair (an *instance *in unlabelled data) is classified as *interacting *or *non-interacting *based on the features describing that pair. A classifier (e.g. a random forest or a support vector machine) is learned from the features of the instances whose interactions are known (labelled data). We use these STRING and GO annotations in building features of protein-pairs. For *labels*, instances that have nonzero value for STRING experimental score are treated as known interactions. Simply sequencing the genome of a bacterium provides genomic context features in STRING. Several bacteria have at least partial annotations available in GO. Of the 1,707 genomes represented in GO, 1,194 have 50-70% of their genomes annotated and only 98 genomes have less than 50% annotated [30].

**Figure 2:**
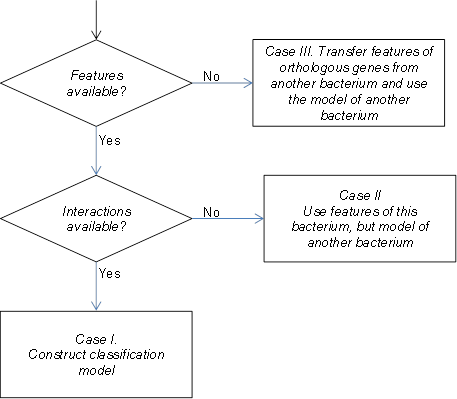
**Approach**. Block diagram showing the approach for different cases of availability of features and interactions in bacterial genomes.

The approach taken for bacterial interactome prediction is shown in block diagram in Figure 2. For a bacterium whose interactome is to be predicted, first we check whether it has annotations present in GO and STRING and a large number of experimentally known interactions in STRING. If both are available (Case I), pairwise features are computed for protein-pairs and a random forest classification model is built to predict hitherto unknown interactions. As shown in Figure 1, typically for many organisms, protein functional and localization features are present even when their interactions may not be available in sufficient numbers to enable training a classification model. In such a case (Case II), our novel approach is to use the computational model built for another organism to predict interactions of the target organism. In order to be able to do this, the feature computation has to be identical between training and "test" protein-pairs (namely, protein-pairs in the target organism). As STRING and GO use a uniform representation and vocabulary, respectively across organisms, it is possible for us to use a uniform representation for pairwise protein features as well. For some other organisms, even the features may not be available for majority of the proteome. In such a case (Case III), first the genes that have orthologs in a well annotated organism are identified and their annotations are used in place of the missing annotations in the target organism. As opposed to other cross-species methods (including STRING predicted scores), this approach has a potential to "predict" interactions even where an interolog may not be available.

In this work, Case I is demonstrated by building Mtb and CD interactomes independently. For Case II, CD is the target organism and is evaluated with computational model of Mtb. For Case III, to demonstrate the advantage of transferring annotations for a computational prediction, the genes in CD that have an ortholog in Mtb are identified and their annotations are set to be 'unavailable' (to compute baseline). The features of protein-pairs in CD that contain these genes are computed by transferring corresponding annotations from Mtb orthologs. Table 1 shows the orthophylogram, which gives phylogenetic distances based on the number of shared orthologs between organisms, for Mtb, CD and also their distance to yeast and human for comparison.

**Table 1:** Orthophylogram distances

	CD	Yeast	Human
Mtb	1-(888 + 868)/(4020 + 4147) = 0.785	1-(735 + 523)/(6717 + 4020) = 0.883	1-(472 + 452)/(22500 + 4020) = 0.9652

CD		1-(374 + 603)/(6717 + 4147) = 0.91	1-(366 + 338)/(22500 + 4147) = 0.9735

Yeast			1-(2206 + 2139)/(22500 + 6717) = 0.8512

Table shows phylogenetic distances between Mtb and CD, and also their distances from yeast and human, based on the number of shared orthologs. Mtb and CD are closer to each other than to yeast or human. The number of orthologs are obtained from KEGG-Orthology [31]*distance *(A, B) = 1 - (number of proteins in A that have orthologs in B + number of proteins in B that have orthologs in A)/(number of proteins in A + number of proteins in B)

### Model generation

To develop a computational model for protein-protein interaction prediction, we assembled a dataset of unique proteins found as a union of those in STRING, GO for Mtb and CD. Seven features were computed for every protein-pair in the proteomes of both the organisms: Gene Ontology features for cellular component, molecular function and biological process, STRING features based on four genomic contexts, namely gene neighbourhood, gene fusion, gene co-occurrence and gene co-expression. Interactions with experimental evidence provided in STRING were used as the gold standard interacting labels. The total data collected for Mtb from the above sources consisted of 4,020 proteins and correspondingly, about 8 million protein pairs, and 3,439 known interacting pairs. CD data consisted of 4,147 proteins, about 8 million protein pairs and 3,414 known interacting pairs. It is common practice to consider randomly generated protein-pairs as non-interacting pairs for training and evaluation of PPIs (after removing any known interactions from this randomly generated set) because there is no authoritative dataset on non-interacting protein-pairs combined with the fact that only one in a thousand or so pairs is expected to interact.

For classification of interacting pairs from non-interacting pairs, we chose to use a random forest classifier as it has previously been evaluated in comparison to Bayesian network models and support vector machines, and has been shown to be most suitable for PPI prediction [32,33]. For each bacterium, a training dataset consisting of 2,500 experimentally known interactions (positive labels) and 120,000 random pairs (negative labels) was created. A blind test set consisting of 500 positive and 150,000 random pairs is created for Mtb and CD separately. The positive to negative ratio in the test sets was chosen to mimic the real world scenario more closely where the chance that a random protein pair is interacting is as low as 1 in 500. For each scenario, a random forest consisting of 14 trees is built using the training set and 3 random features at each branch-point of a tree. Subsequent to evaluation on the test set, the random forest model was used to make predictions for each of the 8 million protein-protein pairs in both Mtb and CD. For cross species training we have used the training set developed for Mtb and evaluated the results by using the test set developed for CD.

### Case I: intra-species interactome prediction

#### Model accuracy on evaluation dataset

The random forest model evaluates each pair and outputs a distribution value ranging between 0 and 1, where larger values correspond to a higher confidence that the pair is interacting. By setting a threshold on this distribution and taking all protein pairs above the threshold, a binary classification value is assigned to each pair. Model accuracy is computed in terms of precision (what percentage of the predicted interactions are known to be true), and recall (what percentage of known interactions are also predicted).

**Figure 3:**
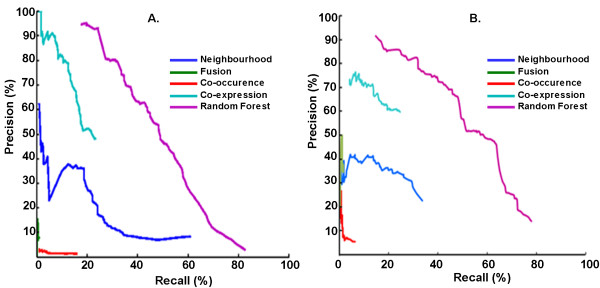
**Precision-recall curves for intraspecies prediction (Case I)**. Precision v/s Recall observed by random forest are compared with that by each STRING scores, which score functional interactions based on genomic context. Random forest predictor achieves a higher recall compared to individual STRING scores for similar values of precision. The "combined score" provided by STRING is also shown to have high false positives in text for *biophysical interactions*. (A) represents the results for training and testing done with Mtb while (B) represents training and testing done with CD.

**Figure 4:**
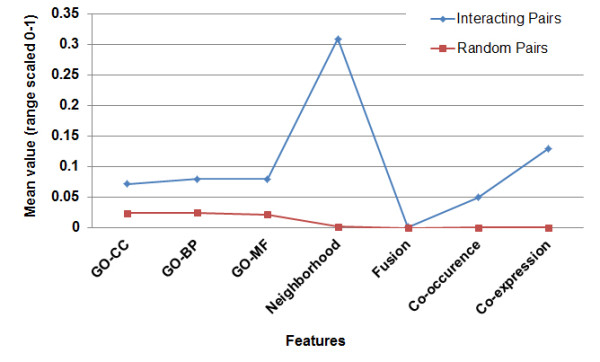
**Mean values of feature elements for interacting and non-interacting pairs**. The figure shows the mean value of each of the 7 features for interacting protein-pairs and for random protein-pairs.

Figure 3 shows the precision-recall curve observed by varying the threshold on the random forest output in predicting Mtb and CD PPIs respectively. For Mtb, by choosing a precision of at least 94%, which occurs at a threshold of 0.9 on random forest output, at least 23% of known interactions are recalled. Correspondingly for CD, precision of at least 90% occurs at a threshold of 0.97 on random forest output, with at least 16% of known interactions being recalled. To compare, on the same evaluation dataset, we computed precision-recall achieved by each of the STRING scores independently. We did not include the STRING combined score and database scores as they include experimental evidence of interaction also. For any chosen precision, the recall is much higher by our method, than by any individual STRING score. The computational model (random forest) views the features in combination with each other, achieving a higher accuracy, even though the mean value of each of the features is well separated between interacting and random pairs, as shown in Figure 4.

#### Predicting new interactions

**Figure 5:**
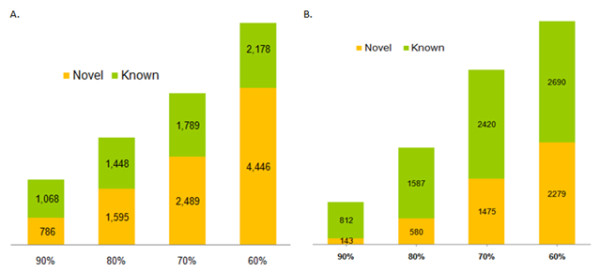
**Number of novel interactions at different levels of precision**. (A) Observed number of novel predictions in the Mtb interactome uncovered at varying level of estimated precisions. For example, at 90% precision, the algorithm uncovers 786 novel interactions and 1,068 known interactions; at expected 80% precision it uncovers 1,595 novel interactions and 1,488 known interactions. The threshold on the random forest output that corresponds to each of the precisions shown in the figure: 0.88 (90% precision), 0.79 (80%), 0.67 (70%) and 0.49 (60%).(B) Observed number of novel predictions in the CD interactome uncovered at varying level of estimated precisions. For example, at 90% precision, the algorithm uncovers 143 novel interactions and 812 known interactions; at expected 80% precision it uncovers 580 novel interactions and 1,587 known interactions. The threshold on the random forest output that corresponds to each of the precisions shown in the figure: 0.98 (90% precision), 0.86 (80%), 0.66 (70%) and 0.58 (60%).

All the protein-pairs in each of the two organisms were evaluated by their corresponding random forest model. The complete list of the protein pairs and their random forest scores are made available for browsing as well as for download at http://severus.dbmi.pitt.edu/TB and http://severus.dbmi.pitt.edu/CD At a very high precision (or selectivity), the number of novel interactions found are 708 for Mtb (with 94% expected precision) and 143 for CD (with 90% expected precision). Additional interactions may be found by lowering the threshold but at the expense of precision. Figure 5 shows number of new interactions found at varying levels of precision for both the organisms.

#### What would the size of the interactome be?

While we are not in a position to make an authoritative estimate of the size of the interactome for the two bacteria, we need an approximate number for the expected number of interactions to put the state-of-the-art into context. It is generally expected that 1 in 500 to 1500 protein-pairs is an interacting pair (based on several estimates of the size of the interactome for different organisms particularly for human and yeast, relative to the size of their proteomes). With this, the expected number of interactions for Mtb, CD, yeast and human would be as shown in Table 2. In a recent study, yeast interactome was estimated to have at least 37,600 interactions, which is within the range of one in 500 to 1500 pairs. In Mtb, there are 4,020 proteins; therefore the interactome would probably have about five thousand to sixteen thousand interactions (see Table 2). Currently, 3,439 interactions are known experimentally. In CD there are 4,020 proteins, and the interactome would probably have about six thousand to seventeen thousand interactions. Currently, 3,414 interactions are known experimentally.

**Table 2:** Known and estimated sizes of interactomes

Organism	Number of proteins	number of pairs	Upper estimate of number of interactions	Lower estimate of number of interactions	Currently known interactions	% relative to larger estimate of interactome size	% relative to smaller estimate of interactome size
Mtb	4,020	8,078,190	16,156	5,385	3,439*	21	62

CD	4,147	8,598,804	17,197	5,732	3,414*	20	60

Yeast	6,717	22,555,686	45,111	15,037	37,000 [34]	82	246

Human	22,500	253,113,750	506,228	168,743	38,000 [35]	8	23

Number of proteins and the size of the interactome are shown for human, yeast, Clostridium difficile and Mycobacterium tuberculosis. Upper estimate of interactome size is obtained by considering that one in 500 random pairs is an interacting pair, and lower estimate is obtained by considering that one in 1500 random pairs is an interacting pair. *These numbers are determined from nonzero STRING experimental scores

**Figure 6:**
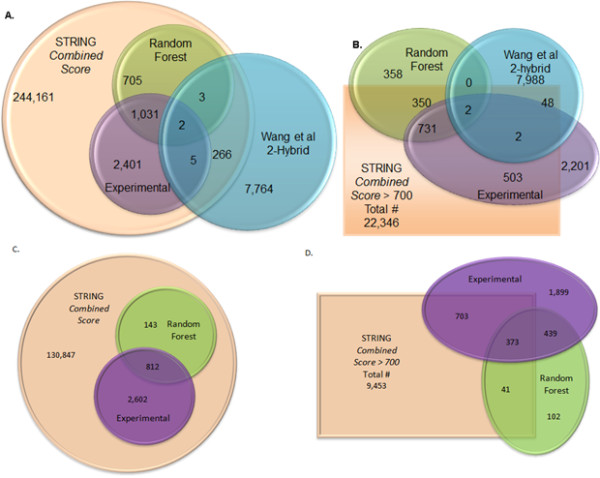
**Overlap of interactome data**. For Mtb, the overlap between interactions predicted by the random forest at 94% precision, and those given by STRING combined score, STRING interactions with experimental evidence and interactions identified by Wang et al by bacterial 2-hybrid method [36]. A. Overlap is shown for entire datasets. For STRING combined score, all interactions with non-zero score combined are considered. B. Similar to (A), scores that have a STRING *combined score *greater than 700 only are shown in the rectangle, while those with less than 700 score are shown outside the rectangle. For CD, the overlap between interactions predicted by the random forest at 90% precision, and those given by STRING combined score, STRING interactions with experimental evidence. C. Overlap is shown for entire datasets. For STRING combined score, all interactions with non-zero score combined are considered. D. Similar to (C), scores that have a STRING *combined score *greater than 700 only are shown in the rectangle, while those with less than 700 score are shown outside the rectangle.

STRING provides a score by integrating information from various other databases, and an overall-score ranging from 0 to 999 obtained as a combination of all these scores. A non-zero score means that at least one of these say that the pair is a functionally related, and possibly interacting. We found that 244,161 Mtb and 127,738 CD protein pairs have a non-zero STRING combined score (Figure 6), which is much larger than the expected size of the interactome (see Table 2). By considering only those pairs that have STRING combined score larger than 700, as is done in practice for network analysis, we found 22,346 Mtb and 7,379 CD pairs. It is crucial to filter non biophysical interactions from these high score pairs. For Mtb, there is another source of interactome data from bacterial 2-hybrid method by Wang et al. They employed the bacterial 2-hybrid method by cloning nearly the entire ORFeome of Mtb and identified 8,040 interactions among 2,907 proteins, with an estimated 57-61% true positives [36].

In order to evaluate the performance of our model, we carried out some analysis of the overlap of our predictions with the widely used STRING based scores as well as with the interactions determined by Wang et al using 2-hybrid technology. Figure 6 shows the overlap between these datasets (our random forest, STRING combined score, STRING experimentally known, and those by Wang et al). In Figure 6A, the overlap is shown with all pairs that have a nonzero STRING combined score. All of the random forest predictions have a nonzero STRING score. What is surprising is that for Mtb, 97% of the bacterial 2-hybrid interactions are not found among non-zero STRING combined score dataset and also have very little overlap with experimentally known interaction data of STRING, despite the fact that their method estimates that their data contains 57-61% true positives [36] In Figure 6B, the overlap is shown considering only those pairs that have a STRING combined score greater than 700. Corresponding numbers for CD are shown in Figures 6C and 6D. What is to be observed here is that majority of the high confidence interactions predicted by random forest and most interactions identified by Wang et al are missed by this threshold on STRING score. What is to be considered as the interactome for these bacteria? We believe that strong computational methods such as random forests can combine information from multiple sources for accurately extending the interactome beyond experimentally-known interactions.

#### Functional significance of interactions predicted for Mtb

The 708 high confidence interactions predicted through our method span across different functional categories. Additional file 1: Table S1 provides a detailed list of these interactions and their GO annotations of molecular function and biological process as well as the KEGG pathways that they belong to [37], where available. 307 of them involve proteins in the same pathway whereas 54 connect two different pathways. For 348 pairs, KEGG pathway is not known for at least one of the two proteins involved in the interaction.

**Figure 7:**
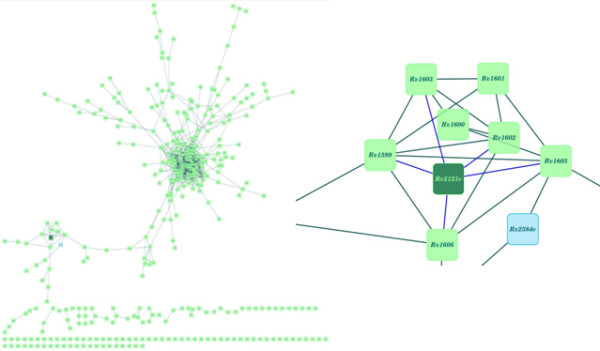
**Network view of predicted interactions**. (A) Complete network of the protein-protein interactions having high confidence. (B) Sub-cluster depicting some of the proteins involved in Histidine biosynthesis pathway. Node for the HisG protein is colored as dark green. Blue node (Rv2584c) is involved in purine biosynthesis.

Some of these are well known interactions in related systems and thus serve as positive controls of the approach. An example of this is described here. Figure 7 shows a network view of only the high-confidence predicted interactions. HisG (Rv2121c) protein is known to play a crucial role in the histidine biosynthesis pathway by catalyzing the first step as an ATP-phosphoribosyl transferase and is also involved in purine biosynthesis [38]. Due to its importance in these biochemical pathways and high degree of conservation amongst the prokaryotes and lower eukaryotes, HisG has been suggested to be a potential drug target [39]. Several high-confidence interactions have been predicted (Figure 7) between HisG protein and other important proteins such as HisH (Rv1602), HisF (Rv1605), HisI (Rv1606), HisD (Rv1599), HisA (Rv1603) which are known to be involved in histidine biosynthesis pathway. These proteins form a well-connected cluster (Figure 7) in the network constructed merely from high confidence interactions predicted here. The regulation of histidine biosynthesis in mycobacteria is believed to be different from that in E.coli [40]. It is also observed from the network of protein-protein interactions that the protein Rv2584c involved in the nucleotide biosynthesis pathway also form a part of this sub-cluster thus highlighting the importance of HisG protein to serve as a potential drug target.

The high-confidence interactions serve as hypotheses to design focussed experiments to study the role of the proteins. Among those predicted, two are described here:

**Rv1538c - Rv3676: **Rv1538c (ansA), L-asparaginase involved in conversion of asparagine to aspartate is predicted to be interacting with Rv3676. The protein Rv3676 is transcriptional regulatory protein and belongs to CRP/FNR family. The functional significance involving orthologs of both the proteins have been studied in *Escherichia coli *[41]. cAMP receptor protein (CRP, ortholog of Rv3676) positively regulates transcription of asparaginase gene (ortholog of Rv1538c). Under the anaerobic conditions, the CRP regulatory protein play major role in facilitating the expression of asparaginase. Similar function is expected to be observed in Mtb by detail inspection of this interaction.

**Rv0350 - Rv3914: **Rv0350 (dnaK) is a chaperone. It is a heat shock protein and is regulated positively by Rv3223c (sigH) protein and negatively regulated by Rv0353 (hspR). This protein is predicted to be interacting with Rv3914 (trxC). The protein Rv3914 is thioredoxin and participates in various redox reactions. In Mtb, it has been observed that under the conditions of oxidative and heat stress, sigH regulates the expression of thioredoxin and dnaK [42].

### Case II: model accuracy on cross species evaluation

**Figure 8:**
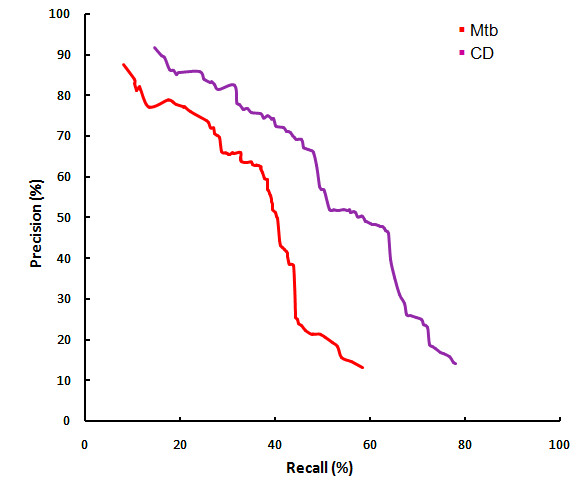
**Precision - recall curve for cross species prediction (Case II)**. Precision v/s Recall observed for cross species evaluation. The CD test set was used for all two evaluations. The purple line shows us the classifier that was trained on the CD features and the red line shows us the classifier that was trained on the Mtb features.

The results of using cross-species computational model are shown in Figure 8. On evaluating the CD features with a random forest model built with features and interactions of Mtb proteome, a precision of at least 88% when 8% of known interactions are recalled (at a threshold of 0.93 on random forest output). That is, for bacterial organisms with no known interactions but with the availability of protein features (3 out of 7 of which are available simply from the genome sequence), at least a partial interactome may be computed by using the model developed for another well studied organism, say Mtb.

### Case III: model generation with ortholog transfer

To find the orthologs for CD in Mtb, we used the KEGG Orthology database [37]. The number of orthologs found between CD and Mtb are given in Table 1, which shows the orthophylogram among Mtb, CD, yeast and human. Three datasets were made from the original CD data used in the cross-species methods. The first was the control, consisting of all CD pairs with their own GO annotations. The second dataset was made to mimic a situation in which few GO annotations were available. This was done by taking the control and removing all GO annotations for any genes with orthologs in Mtb. Of all the protein pairs used in the testing and training sets, 119,562 pairs had their GO annotations removed. The final dataset was a modification of the second set. This involved inserting the GO annotations of the orthologs in Mtb to their corresponding protein pairs in the dataset. A total of 79,331 GO annotations were transferred from Mtb to CD. This number falls short of the 119,562 values that were removed in the process of creating the second dataset. This is because the GO annotation coverage of Mtb is 71.8%, so some orthologs in Mtb are also without annotations.

**Figure 9:**
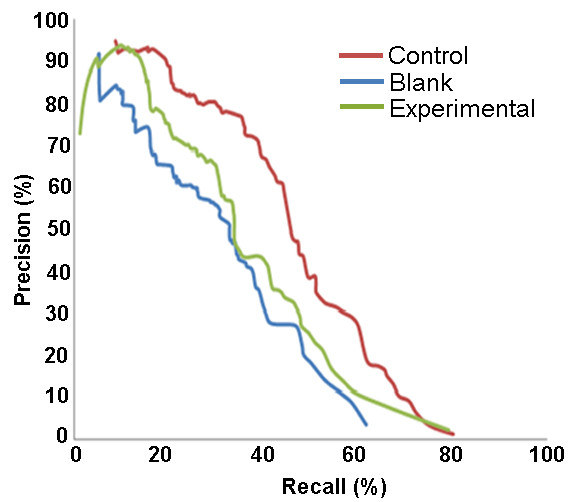
**Precision and recall for ortholog transfer (Case III)**. The data that used orthologous Gene Ontology values in substitution (experimental) consistently performed better than the data with removed value (blank), and managed to reach an accuracy equal to our control, which would be having all available data for CD.

Traditional precision and recall plots were used to compare the performance associated with the three models. From Figure 9, it can be seen that the model built on the data with the missing features (second dataset) had the lowest value of precision and also the lowest performance. The model built on the dataset that was constructed by transferring missing features from orthologs (third dataset) in Mtb had higher values of precision, for similar values of recall than the model trained on the second dataset. Thus transfer of features from orthologs from a well annotated organism, enriched the training data, due to which we were able to build a better model.

In the transfer of GO annotations with orthologous genes, a consistent improvement was observed (see Figure 9). The use of ortholog transfer was between 8-10% more accurate than the data without gene annotations across the board. In the upper ranges of the model's accuracy, it managed to achieve accuracy equal to the control method.

## Conclusions

We presented three scenarios of availability of proteome annotations for bacteria and presented approaches for rapid development of a computational system for bacterial interactome prediction, even in scenarios where little or no interaction or feature information associated with protein-pairs is available, resulting in significant savings in cost, time and effort. The methods have been demonstrated with two candidate bacterial proteomes, namely that of *Mycobacterium tuberculosis *(Mtb) and *Clostridium difficile *(CD). For the first scenario, we developed random-forest classifiers which filtered functional linkages obtained from the STRING database for Mtb and CD, by adding gene ontology features to the STRING features. We could identify potential biophysical interactions amongst these linkages with a very high precision using the classifier. In the second scenario, we demonstrated *computational transfer *across species by predicting CD interactions using the classifier trained over Mtb interactome. This work demonstrates that with the availability of a comprehensive interactomes for a few organisms, interctomes for other organisms may be predicted not only bioinformatically but also by transfer of computational models. We also demonstrated cross-species information may be used not only for interolog transfer, but also for computational prediction of interactions where only one of the two proteins in the pair may have an ortholog, or when both pairs may have an ortholog but not necessarily their interaction. These methods can be used for bacterial proteomes for which few features and/or interactions are available.

## Availability

Novel interactions have been identified for both Mtb and CD, and have been made available online at http://severus.dbmi.pitt.edu/TB and http://severus.dbmi.pitt.edu/CD

## Methods

### Feature-set description

The features that were used in the computational prediction are listed in Table 3, and are described in detail below:

**Table 3:** Features of protein-pairs used to in computational model

Features used	Attribute type
Gene Neighborhood score, obtained from STRING	Real valued, ranges from 0-999

Gene Fusion score, obtained from STRING	Real valued, ranges from 0-999

Gene Co-occurrence score, obtained from STRING	Real valued, ranges from 0-999

Gene Co-expression score, obtained from STRING	Real valued, ranges from 0-999

GO Cellular component based feature	Real valued, ranges from 0-0.5

GO: Molecular function	Real valued, ranges from 0-0.5

GO: Biological process	Real valued, ranges from 0-0.5

Each protein-pair is presented with seven features, four of which are obtained directly from STRING database, and three of which are computed based on GO annotations

### Gene ontology features

Gene Ontology (GO) provides a controlled vocabulary to describe gene products, and the relations among the terms in the vocabulary. The terms are arranged in the form of a directed acyclic graph (DAG), with three major branches from the root corresponding to molecular function (MF), cell component (CC) and biological process (BP). Annotations of gene products by these terms are created by members of the GO project by curating literature. When the DAG is traversed away from the root, the terms describe more and more specific characteristics of the gene product, whereas when traversing towards the root they become more general. For some genes only broader information is known (e.g. molecular function is *hydrolase activity*), whereas for some other proteins more specific information may be known (e.g. molecular function is *hydrolase activity, acting on ester bonds*). For use in some applications such as the current work, a smaller subset of vocabulary called *GO Slims *is available, which maps lower level (more specific) GO terms to a higher-level *Slim *category. In this work, the GO Slim annotations have been used. All GO terms associated with a protein are mapped to the GO Slim categories that were their closest ancestor in the GO DAG. For instance, Endoplasmic Reticulum (GO:0005783) is the closest ancestor of the term ER proteasome core complex, alpha-subunit complex (GO:0031605) and is also present in the GO-Slim as a broad cellular localization category. Thus GO:0031605 is mapped on to the broader cellular-localization term of Endoplasmic Reticulum. Using this approach, we assigned GO Slim categories to every protein, based on their GO term annotations. For molecular function and biological process, we made use of the GO-Slim OBO files provided by the gene ontology website, but for cellular localization, we used the GO Slim categories that we had previously created manually. The complete mapping of specific GO terms to each of the broader GO Slim terms or categories is provided for download at http://severus.dbmi.pitt.edu/TB/.

Most approaches for protein-protein interaction prediction encode the fact that a protein often shares similar features as its interacting partners. In the context of GO annotations, it is assumed that a protein would likely localize to the same cellular component destinations, or partake in the same or similar biological process, or perform comparable molecular functions as that of its interacting partners. By this reasoning often, similarity scores between GO terms of the protein-pair are encoded as a feature for PPI prediction. However, these methods do not take into consideration the *possible *correlations that exist between different GO categories of interacting partners. For example, it is often observed that a protein having some interacting partners in the extracellular region and some in the cytoplasm, is restricted to reside in the cell membrane, and not necessarily in the same localization as its interacting partners. Thus instead of using GO similarity as a feature, we decided to incorporate correlations between GO categories of interacting proteins in the training data, as a feature for PPI prediction.

A contingency table of term associations is computed showing number of co-occurrences of each pair of GO-slim terms that occur in known interactions (i.e. one GO slim term is associated with one protein, while the other term is associated with its interacting partner) in the training data. This is done separately for CC, MF and BP terms. From this, the feature value for a pair of proteins *P_i _*and *P_j _*having GO slim terms *i *and *j *is computed as the odds ratio *(N_ij_/N_i_N_j_) *where N_ij _represents the value from contingency table corresponding to GO slim terms *i *and *j*. N_i _and N_j _represent individual frequency of occurrence of terms *i *and *j *amongst all annotations of all interacting proteins in the training data. This is done individually for CC, MF and BP, resulting in three features. In the use of orthologous GO annotations, the contingency tables were made from the Mtb interactome, as we would assume that had CD not been thoroughly researched, a CD contingency table would not have enough information to properly represent the co-occurrence of Gene Ontology annotations.

### STRING features and interactions

STRING database provides experimentally known interactions and pairs that are predicted to have functional linkages. It assigns a score to a protein-pair indicating the functional linkages that are present between them. STRING scores derived from gene neighbourhood, gene co-occurrence, gene co-expression and gene fusion were taken as provided by the database. These form the 4 features representing a protein-pair in our dataset. Additionally, the "combined score" provided by STRING was also downloaded for comparison with other methods but is not in our model. All protein-pairs with a non-zero STRING experimentally-derived score were considered to be interacting proteins.

### Orthologous genes

Orthologous genes were obtained from the KEGG Ortholog database [31].

### Random forest

The Weka package (Weka 3.6.0)'s Random Forest implementation was used to build the classifier model from the training data [43].

### Evaluation metrics

We used standard precision and recall plots to evaluate the model's performance. For varying thresholds, both precision and recall associated with the model was recorded, and a graph showing the recall on the x-axis and the precision on the y-axis was constructed. Thus, when different models are to be compared, one can do so, by observing their recall values for similar values of precision or vice-versa and see if for all values of precision, there is a general trend towards improvement in recall.

## Competing interests

The authors declare that they have no competing interests.

## Authors' contributions

MG designed and supervised the work. SA developed the Mtb interactome. RM and NS provided the biological context to the project. AK, AG and AH carried out the cross-species and ortholog transfer evaluations during their summer internships in 2011. Manuscript is written by SA, RM and MG with inputs from all the authors. All authors read and approved the manuscript.

## Supplementary Material

Additional file 1 **The file gives the GO annotations and KEGG pathway annotations, where available, for the two proteins in each of the 709 newly predicted interactions**. The first sheet shows all the annotations, whereas the remaining three sheets show GO molecular function, biological process and KEGG pathways, respectively. In these three sheets, the rows are colored respectively in blue, green or grey depending on whether the two proteins in a pair have different annotations, same annotations, or whether the annotations are not known for either of the proteins. Click here for file
